# A Two-Dimensional Simulation Model of the Bicoid Gradient in *Drosophila*

**DOI:** 10.1371/journal.pone.0010275

**Published:** 2010-04-21

**Authors:** Jingyuan Deng, Wei Wang, Long Jason Lu, Jun Ma

**Affiliations:** 1 Division of Biomedical Informatics, Cincinnati Children's Hospital Research Foundation, Cincinnati, Ohio, United States of America; 2 Division of Developmental Biology, Cincinnati Children's Hospital Research Foundation, Cincinnati, Ohio, United States of America; 3 Department of Biomedical Engineering, University of Cincinnati, Cincinnati, Ohio, United States of America; 4 Department of Chemical Engineering, University of Cincinnati, Cincinnati, Ohio, United States of America; University of Texas MD Anderson Cancer Center, United States of America

## Abstract

**Background:**

Bicoid (Bcd) is a *Drosophila* morphogenetic protein responsible for patterning the anterior structures in embryos. Recent experimental studies have revealed important insights into the behavior of this morphogen gradient, making it necessary to develop a model that can recapitulate the biological features of the system, including its dynamic and scaling properties.

**Methodology/Principal Findings:**

We present a biologically realistic 2-D model of the dynamics of the Bcd gradient in *Drosophila* embryos. This model is based on equilibrium binding of Bcd molecules to non-specific, low affinity DNA sites throughout the *Drosophila* genome. It considers both the diffusion media within which the Bcd gradient is formed and the dynamic and other relevant properties of *bcd* mRNA from which Bcd protein is produced. Our model recapitulates key features of the Bcd protein gradient observed experimentally, including its scaling properties and the stability of its nuclear concentrations during development. Our simulation model also allows us to evaluate the effects of other biological activities on Bcd gradient formation, including the dynamic redistribution of *bcd* mRNA in early embryos. Our simulation results suggest that, in our model, Bcd protein diffusion is important for the formation of an exponential gradient in embryos.

**Conclusions/Significance:**

The 2-D model described in this report is a simple and versatile simulation procedure, providing a quantitative evaluation of the Bcd gradient system. Our results suggest an important role of Bcd binding to non-specific, low-affinity DNA sites in proper formation of the Bcd gradient in our model. They demonstrate that highly complex biological systems can be effectively modeled with relatively few parameters.

## Introduction

Development is a robust process that must achieve a precise and reproducible outcome despite inevitable variations among individual embryos [1], [2], [3], [4]. One such variation is the size of embryos. In *Drosophila*, patterning along the anterior-posterior (A–P) axis exhibits scaling properties such that embryonic structures are patterned in proportion to embryo length [5], [6], [7], [8]. Bicoid (Bcd), a morphogenetic protein in *Drosophila*, is responsible for instructing patterning along the A–P axis [9], [10], [11]. How developmental scaling is achieved is a subject of intense interest [12], [13], [14]. Several models have been proposed to explain scaling along the A–P axis in *Drosophila* but they currently remain at a theoretical level [15], [16], [17], [18], [19], [20], [21], [22]. For example, it was proposed recently that, theoretically, scaling could be achieved through a cytoplasmic streaming field [21], but the proposed streaming has not yet been observed experimentally.

Recent experimental studies have significantly advanced our knowledge on Bcd gradient behavior in *Drosophila* embryos. These new findings represent an important foundation for developing and evaluating models of Bcd gradient formation. In particular, a recent live-imaging study demonstrated that Bcd concentrations inside individual nuclei exhibit a striking stability even though the number of nuclei is doubling after each nuclear division in early embryos [23], [24]. These findings are important because they suggest that the positions of specific nuclear Bcd concentration thresholds are maintained even though the embryo itself, during this critical period of development, is undergoing an exponential growth in terms of the number of nuclei. In another study using stained embryos, we observed that the Bcd gradient itself exhibits properties of scaling [7]. In particular, the amount of Bcd in the anterior is correlated with embryo size. Furthermore, Bcd intensity is more precise when measured as a function of normalized A–P position than without such normalization. In embryos from *staufen* (*stau*) females, a loss of Bcd gradient scaling can directly explain the scaling defect of its target expression boundary. These findings suggest that the scaling properties of the Bcd gradient itself are important for scaled patterning along the A–P length in early embryos.

According to a widely-held view [25], Bcd gradient formation can be described as a dynamical process of localized protein production and embryo-wide diffusion and degradation. To fully describe and understand the Bcd gradient system, it is necessary to have a realistic model that considers the geometry of the embryo, the changing diffusion media within which the Bcd gradient is formed, and key features of the *bcd* mRNA from which Bcd is produced. For example, unlike what is assumed in most of the current models, the maternally deposited *bcd* mRNA is not restricted to a single point at the anterior tip [26], [27], [28], [29]. Instead, it forms a cloud-like shape in early embryos with its own volume, shape, density and location, properties that affect where Bcd molecules are produced in the embryo and, thus, how the gradient is formed. How the distribution of *bcd* mRNA may affect the shape and formation of the Bcd gradient remains an important question [30]. This question is further highlighted by a recent report that challenged the diffusion model [31]. Based on the observed dynamic distributions of *bcd* mRNA in early embryos, it was suggested that the shape and formation of the Bcd protein gradient are dictated by *bcd* mRNA distributions.

In this report, we develop a biologically realistic 2-D model to simulate the formation of the Bcd gradient in *Drosophila* embryos. It allows us to evaluate, among other things, the effect of *bcd* mRNA redistribution on Bcd gradient formation, suggesting that, in our model, Bcd protein diffusion is important for the formation of an exponential protein gradient. We also present simulation results that recapitulate several other important features of the Bcd gradient, including the experimentally observed scaling properties of the Bcd gradient and the stability of nuclear Bcd concentrations between different nuclear cycles.

## Results and Discussion

### Model

A primary goal of our current study is to establish a biologically realistic model that simulates the dynamics of Bcd protein distribution in early *Drosophila* embryos, focusing on evaluating the effects of the changing diffusion media within which the Bcd gradient is formed and the molecular properties of *bcd* mRNA from which Bcd protein is produced. Since stochastic properties of the Bcd gradient system have been extensive investigated recently [32], [33], [34], they are not the focus of our current work. Here, the fluctuation in the number of Bcd molecules is averaged within a finite volume in our simulations [35], [36], [37], [38] to reveal the “average” properties of the Bcd gradient. In our model, we consider two forms of Bcd molecules: free-diffusing molecules, B_free_, that are in the cytoplasm, and immobile molecules, B_bound_, that are bound to low-affinity DNA sites inside the nuclei. The binding/unbinding behavior of Bcd to these DNA sites is characterized by the rate constants *k_1_* and *k_2_*, respectively, and expressed as:
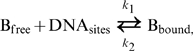
(1)At equilibrium, the ratio of bound to free Bcd molecules is determined by the association constant *K_A_* = *k_1_*/*k_2_*:
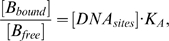
(2)

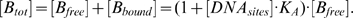
(3)Since only the unbound Bcd molecules in the cytoplasm can diffuse freely in our model, the effective diffusion constant *D_eff_* can be expressed as:
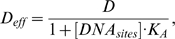
(4)where *D* is the diffusion constant for free-diffusing Bcd molecules inside the cytoplasm.

**Figure 1:**
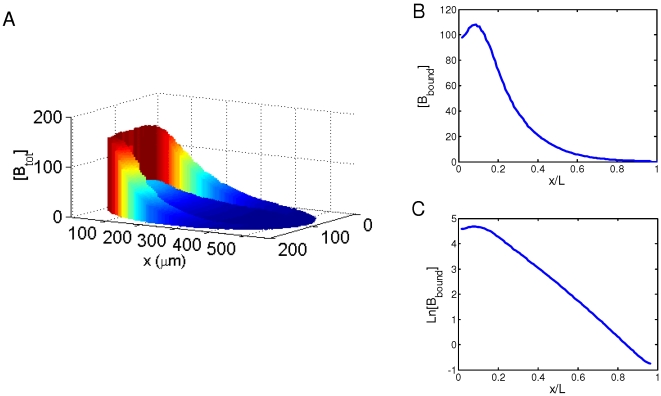
Simulated Bcd distributions in a *Drosophila* embryo. A. A simulated embryo at nuclear cycle 14 showing the local total Bcd concentration [B_tot_] (arbitrary units). The A–P position is shown as absolute distance *x* (in µm) from the anterior. The ratio of total Bcd molecules in the cortical layer to those in the inner part of the embryo is 1.88 at nuclear cycle 14 (see text for further details). B. A plot of local DNA-bound Bcd concentration [B_bound_] (arbitrary units) within the cortical layer as a function of fractional embryo length *x*/*L*. In this and other figures presented in this report, [B_bound_] at each A–P position represents the mean [B_bound_] value of all cubes within the cortical layer of the embryo at that A–P position. C. Same as B except [B_bound_] is on ln scale. Linearity of ln[B_bound_] indicates an exponential Bcd protein gradient; see text and Fig. 2 legend for more information about Adjusted *R* ^2^ values to further evaluate the quality of exponential fitting of the simulated data.

In our 2-D model, we simulate the mid-coronal slice of the *Drosophila* embryo, where Bcd molecules are distributed. An ellipse is selected to represent the boundary of the embryo slice, with major axis *a_major_* and minor axis *b_minor_*. The shape of the ellipse is similar to the mid-coronal section of a typical wt embryo with *a_major_* = 550 µm and a *b_minor_*/*a_major_* ratio of 1/3. A 2-D mesh grid system is used to divide the embryo slice into uniform cubes that are arranged on the coronal 2-D plane, each with a volume of *Δx* ^3^ µm^3^. We performed simulations with *Δx* = 2 and 5 µm, and obtained consistent results based on values for the three established criteria that quantify Bcd gradient properties (see below); *Δx* ^3^ = 5^3^ µm^3^ represents a cube volume that approximates the volume of a nucleus in early embryos [32]. The behavior of Bcd molecules within each cube, described as concentrations, is determined by both its location and developmental stage (i.e., nuclear cycle). The effective diffusion constant of Bcd molecules is a function of both nuclear cycle and the location. We use *D*(*c*;*i*,*j*) to denote the effective diffusion constant in cube (*i,j*) at the *c*th nuclear cycle. During nuclear cycles 1–9, the entire embryo is treated as a homogeneous medium filled with cytoplasm, in which the nuclei are evenly distributed. The number of nuclei during this period is limited (2^8^ = 256 at nuclear cycle 9 [39]). Thus, the influence of nuclei on Bcd diffusion is ignored in our simulations and *D*(*c*;*i*,*j*) is selected to be *D* for all cubes within the embryo boundary during nuclear cycles 1–9. At the onset of nuclear cycle 10, all nuclei become located in the cortex of the embryo in our model, forming the cortical layer (an estimated ∼30 µm is used in our main model shown in Figs. 1–5) where the nuclear number continues to double after each nuclear division, and leaving the bulk of the embryo as the nucleus-free inner part (the yolk). Upon the formation of the cortical layer, the diffusion behaviors of Bcd molecules in these two distinct homogeneous media are different in our model. *D*(*c*;*i*,*j*) remains to be *D* for all cubes located within the nucleus-free inner part of the embryo. For cubes within the cortical layer, the doubling of nuclei number after each nuclear division leads to the doubling of the concentration of low-affinity DNA sites for Bcd, with the effective diffusion constant of Bcd molecules expressed as:
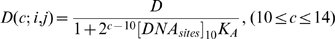
(5)where [DNA_sites_]_10_ is the concentration of low-affinity DNA sites for Bcd within the cortical layer at nuclear cycle 10.

**Figure 2 pone-0010275-g002:**
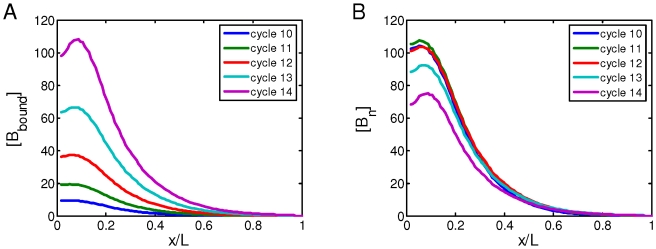
Stability of nuclear Bcd concentrations. A. A plot of [B_bound_] within the cortical layer as a function of *x*/*L*, at nuclear cycles 10–14. B. Same as in A, except now showing nuclear Bcd concentrations ([B_n_]
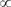
[B_bound_]/nuclear number; all in arbitrary units) within the cortical layer at nuclear cycles 10–14. While [B_bound_] increases after each nuclear division (as seen in panel A), [B_n_] remains stable in the simulated embryo (as seen in panel B). The length constant *λ* of the simulated [B_bound_] within the cortical layer at nuclear cycles 10–14 is: 72, 79, 85, 89 and 92 µm, respectively. The Adjusted *R* ^2^ values of exponential fitting of simulated [B_bound_] during nuclear cycles 10–14 are, respectively: 0.9985, 0.9988, 0.9992, 0.9996 and 0.9998 for the fitting region of *x*/*L* = 0.2 to 0.7; and 0.9454, 0.9470, 0.9456, 0.9402, and 0.9296 for the fitting region of *x*/*L* = 0 to 0.7.

**Figure 3 pone-0010275-g003:**
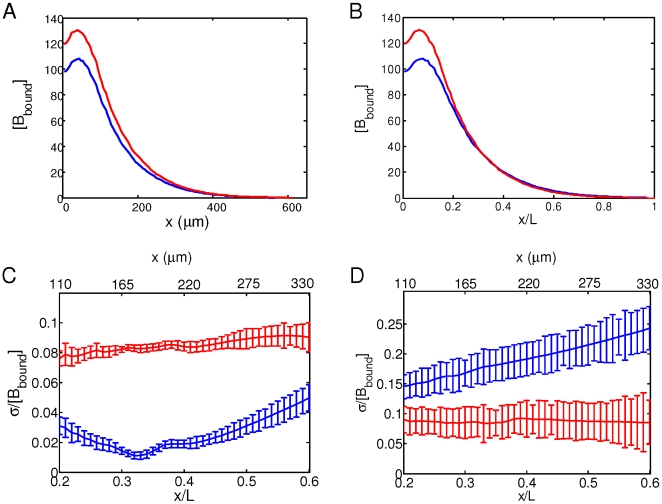
Scaling properties of the Bcd gradient. A. A plot of [B_bound_] (arbitrary units) within the cortical layer of two simulated embryos with distinct lengths (550 µm and 600 µm) as a function of absolute distance *x* (in µm) from the anterior. In this simulation, the amount of *bcd* mRNA is correlated with the volume of the embryo, i.e., the aggregate *J* value is correlated with embryo volume. B. Same as in A, except with the use of normalized A–P position *x*/*L*. Note the convergence of the two profiles. C. Noise (standard deviation divided by the mean) of [B_bound_] within the cortical layer in 50 simulated embryos, plotted as a function of either absolute distance *x* (in µm) from the anterior (red, upper scale) or normalized A–P position *x*/*L* (blue, lower scale). Error bars are from bootstrapping. The lengths of simulated embryos are variable and follow normal distribution with a mean of 550 µm and standard deviation of 20 µm. In this plot (as in panels A and B), the total amount of *bcd* mRNA is correlated with embryo volume. D. Same as C, except that there is no correlation between the total amount of *bcd* mRNA and embryo volume. Color codes are the same as in C.

**Figure 4 pone-0010275-g004:**
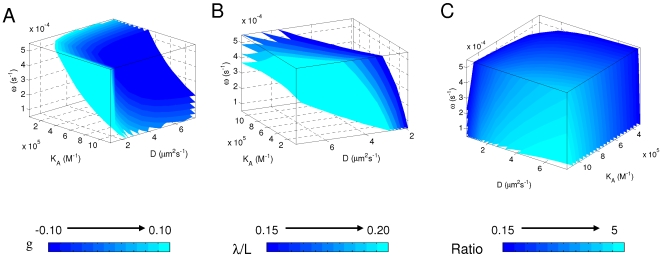
Systematic evaluation of parameter values on system behavior. 3-D presentation of parameter space satisfying each of the three criteria: [B_n_] stability as measured by *g* (panel A), gradient shape as measured by length constant *λ* at nuclear cycle 14 (panel B), and cortical enrichment as measured by the ratio of total Bcd molecules in the cortical layer to those in the inner part of the embryo at nuclear cycle 14 (panel C). In our systematic sampling, we tested all possible parameter value combinations (within the tested ranges at the tested increments) and the simulated results are evaluated according to the three criteria. Also see Fig. S2 for presentations showing the effects of changes in individual parameters while holding the other two at set values used in the main model simulation.

**Figure 5:**
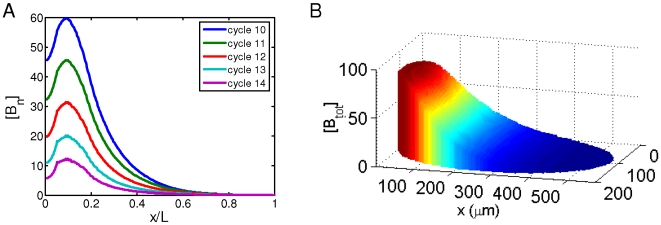
The effects of *K_A_* on simulated Bcd gradient properties. A. A plot of [B_n_] at nuclear cycles 10–14 in a simulation in which *K_A_* = 10^7^ M^−1^. This figure is the same as Fig. 2B, except the difference in *K_A_* values used in simulations (*K_A_* = 5×10^5^ M^−1^ for Fig. 2B). Note the instability of [B_n_] profiles between different nuclear cycles (*g* = −0.3557) and a reduction in length constant (*λ/L* = 0.12 at nuclear cycle 14) in this simulation. B. A simulated embryo showing [B_tot_] at nuclear cycle 14. This figure is the same as Fig. 1A except that different *K_A_* values are used in simulations (*K_A_* = 10^4^ M^−1^ for this figure and *K_A_* = 5×10^5^ M^−1^ for Fig. 1A). Note a lack of cortical layer enrichment of Bcd molecules in this simulation, with a ratio of total Bcd molecules in the cortical layer to those in the inner part of the embryo, *Ratio* = 0.6328.

In our simulations, we assign a status to each cube in our mesh grid system depending on its center coordinate and developmental time: the cortical layer (I) and the inner part (II); prior to the formation of the cortical layer, all cubes within the embryo boundary are treated as status II as discussed above. We further assign status III to cubes that contain *bcd* mRNA and, thus, produce Bcd protein at a constant rate *J* (mol/s). In the main model simulations (Figs. 1–5), the *bcd* mRNA sphere is modeled as a disc (orthographic projection) inside the simulated embryo slice, with a uniform density, a radius of 45 µm and a center coordinate of (75 µm, 0); the shape, location and amount of *bcd* mRNA remain constant at all nuclear cycles in these simulations. The *J* value is identical for all cubes that contain *bcd* mRNA; thus the effective Bcd production rate for the entire embryo is the aggregate *J* (i.e., individual cube *J* value times cube number). At time *t*, Bcd molecules within each cube are in equilibrium with the low-affinity DNA sites, and we use the Euler forward method (
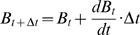
) to numerically calculate [B_tot_] in cube (*i*,*j*) at time *t*+Δ*t* as a result of protein diffusion to and from its four immediate neighbors and position-independent protein degradation:
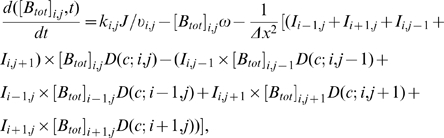
(6)where *υ_i,j_* is the volume of cube (*i,j*) and *ω* is the degradation rate of Bcd molecules. *I_i,j_* and *k_i,j_* are binary values (0 or 1) depending on the status of the cube:
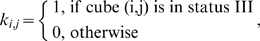
(7)and
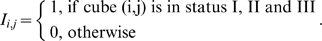
(8)In our simulations we used Δ*t* = 0.2s; we also tested Δ*t* = 0.01, 0.1, 0.5 and 1.0s and obtained consistent results based on the values for the three established criteria to quantify simulated Bcd gradient behavior (see below), indicating that our solutions are numerically stable.

The developmental time in all our simulations is based on experimental observations [24], [39]. In our simulations, *t* = 0 represents the time of egg deposition and our simulations end at 15 minutes into nuclear cycle 14. Nuclear cycles 10–14 start at 81, 95, 110, 125 and 140 minutes after egg deposition, respectively. The Bcd concentration profiles for nuclear cycles 10–14 shown in this report represent simulated data at 94, 109, 124, 139 and 154 minutes after egg deposition unless otherwise stated. The parameter values used in our simulations are either calculated based on estimates (for [DNA_sites_]_10_, see below) or chosen through a systematical sampling within the biologically reasonable ranges to yield Bcd gradient properties that satisfy three established criteria (see below). In our main model (Figs. 1–5), parameter values are as follows unless stated otherwise: *D* = 2 µm^2^s^−1^, *ω* = 0.00005 s^−1^, *K_A_* = 5×10^5^ M^−1^, and [DNA_sites_]_10_ = 3×10^−7^ M. [DNA_sites_]_10_ is calculated based on the following estimates in our main model: there are 512 nuclei at nuclear cycle 10, each diploid nucleus contains ∼2.4×10^8^ bp euchromatic DNA [40] with an estimated ∼2×10^6^ copies of 5′-TAAT-3′ DNA sites that represent the low-affinity DNA sites for Bcd, and the cortical layer volume is ∼5.8 nl within which all the low-affinity DNA sites are distributed homogeneously for simulation convenience. The parameter value for aggregate *J* is set arbitrarily in our simulations; this value does not affect the three criteria (see below) that we establish to quantify the Bcd gradient behavior. In our simulation results, nuclear Bcd concentrations [B_n_] are calculated from the local DNA-bound Bcd concentrations [B_bound_]. Since the nuclear volume remains a constant between different nuclear cycles in our main model (Figs. 1–5), [B_n_]
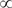
[B_bound_]/nuclear number.

We establish three different criteria to quantify the behavior of the simulated Bcd gradient. First, we measure the stability of nuclear Bcd concentration [B_n_] during nuclear cycles 10–14 by computing the space-dependent relative change of [B_n_] between the two successive nuclear cycles and then average the differences over space and nuclear cycles: 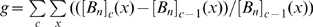. We consider a [B_n_] gradient profile to be stable if |*g*|<0.1 [24]. Second, we measure the enrichment of Bcd molecules within the cortical layer using the ratio of total Bcd molecules within the cortical layer to those within the inner part of the embryo. We regard Bcd molecules to be enriched in the cortical layer if *Ratio*>1.5 at nuclear cycle 14. Third, we evaluate the shape of the Bcd gradient using its length constant *λ*, and we assume a gradient to be properly shaped if *λ/L* has a value between 0.15 and 0.2 [5], [7], [23]. To evaluate how well a simulated Bcd profile in a given region of the embryo fits an exponential function, we also calculate Adjusted *R*-square (Adjusted *R* ^2^) values [41]. Curve Fitting Tool in Matlab was used for fitting the exponential function 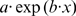.

### Basic 2-D simulation results of main model

Fig. 1A represents a simulated 2-D embryo at nuclear cycle 14 showing the local total Bcd concentration [B_tot_]. These simulation results realistically recapitulate the biological system and reveal several important features that are consistent with experimental observations. First, consistent with experimental observations [5], [7], [9], [24], [42], Bcd is concentrated within the cortical layer (Fig. 1A). Within this layer, the local DNA-bound Bcd concentration forms an exponential gradient (see Fig. 1B,C) with a length constant λ of 92 µm, in agreement with experimentally measured values [5], [7]. Second, unlike other models in which Bcd production is restricted to a single point at the anterior resulting in a Bcd concentration gradient that follows an exponential function throughout the entire A–P length, our simulated results realistically reproduce the experimentally observed “deviation” at the anterior part of the embryo, a region where Bcd concentrations are known not to follow the exponential function ([5], [7], [42]; see Fig. S1 for a comparison between simulated data and experimental data). While the simulated data in the region of *x*/*L* = 0.2 to 0.7 exhibit an excellent exponential fit (Adjusted *R* ^2^ = 0.9998; same value also obtained for the fitting region of *x*/*L* = 0.2 to 1), the exponential fitting becomes significantly worse when the simulated data points from the most anterior part of the embryo are included (Adjusted *R* ^2^ = 0.9296 and 0.9461 for the fitting regions of *x*/*L* = 0 to 0.7 and *x*/*L* = 0 to 1, respectively). The observed anterior “deviation” of the Bcd gradient profiles in our 2-D model is due to the size and location of *bcd* mRNA (see Fig. S1 for 2-D simulation results where *bcd* mRNA is strictly located at the anterior tip).

### Stability of Bcd gradient at different nuclear cycles

One of the most striking features of the Bcd gradient dynamics is the stability of nuclear Bcd concentrations during nuclear cycles 10–14 [24]. To specifically evaluate our model on this critical feature, we simulated Bcd gradient formation and analyzed Bcd concentration profiles at distinct nuclear cycles. We plotted two separate profiles: local DNA-bound Bcd concentration [B_bound_] and nuclear Bcd concentration [B_n_], which are shown in Fig. 2A and B, respectively. In these figures, Bcd concentration profiles at nuclear cycles 10–14 are shown. Our results demonstrate that, even as the nuclear number doubles after each nuclear division during this period, the profiles of nuclear Bcd concentrations [B_n_] remain stable (Fig. 2B). Consistent with the live-imaging data [24], our simulated [B_n_] profiles maintains <10% variation between nuclear cycles 10–14 throughout the entire A–P length. Similar to nuclear cycle 14 (Fig. 1B,C), the simulated [B_n_] profiles at other nuclear cycles also follow an exponential function with a length constant *λ* consistent with experimental values and exhibit the proper anterior “deviation” from an exponential function (see Fig. 2 legend for details).

### Scaling properties of the Bcd gradient

As suggested by our recent studies [7], the scaling properties of embryonic patterning along the A–P axis can be directly traced to the scaling properties of the Bcd gradient itself. To determine whether our model can recapitulate scaling properties of the Bcd gradient, we conducted two different analyses. In our simulations, we assume that the amount of *bcd* mRNA is correlated with the embryo volume. This is a reasonable assumption (but remains to be tested experimentally) if the amounts of maternally-deposited components, including *bcd* mRNA, are proportional to the egg volume, i.e., the concentrations of various maternal components remain as constants among individual eggs even though the total maternally-deposited contents (i.e., egg volume) can vary between eggs. In our first analysis, we simulated two individual embryos that have distinct lengths (550 µm and 600 µm). Fig. 3A and B show the local DNA-bound Bcd concentration profiles at nuclear cycle 14 expressed as a function of, respectively, absolute distance from the anterior *x* (in µm) and fractional embryo length *x*/*L*. Our results show that, while these two profiles in the anterior and middle portions of the embryo are apart from each other in the *x* plot, they converge in the *x*/*L* plot, most notably in the broad, mid-portion of the embryo. When the same *bcd* mRNA amount was applied to these two embryos, such a convergence was not observed (data not shown). In a second analysis, we simulated a group of 50 embryos that differ in size (see Fig. 3 legend). We calculated [B_bound_] noise (standard deviation divided by the mean) at nuclear cycle 14 for these embryos as a function of either *x* or *x*/*L*. Our results (Fig. 3C) show a significantly lower [B_bound_] noise in *x*/*L* than in *x*, a finding that is fully consistent with experimental data ([7]; note that all of the simulated [B_bound_] noise levels are lower than experimental values because parameters, except *L*, do not have variations in our simulations). In contrast, when there is no correlation between the *bcd* mRNA amount and embryo size in our simulations, [B_bound_] noise actually became higher as a function of *x*/*L* than of *x* (Fig. 3D). These two sets of analyses demonstrate that, with a simple assumption that the amount of *bcd* mRNA deposited into an egg is proportional to egg volume, our model can readily reproduce the experimentally observed scaling properties of the Bcd gradient.

### Evaluation of parameter values on system behavior

Although the precise values for the parameters *D* and *ω* remain either controversial or unknown at this time [24], [43], [44], the values chosen in our main model simulations described thus far were selected from a systematic sampling to yield Bcd gradient properties consistent with experimental observations as evaluated by the three criteria established above. Here, we present our sampling results in Fig. 4, with individual panels showing the identified parameter space satisfying each of the three criteria. While the space satisfying the cortical enrichment criterion is relatively broad (Fig. 4C), the space that satisfies the other two criteria is narrower (Fig. 4A, B), particularly for the gradient shape criterion *λ* (Fig. 4B). To further illustrate the effects of individual parameters on model performance, we also plot the criterion values as a function of each of the three parameters while holding the other two parameters at set values used in the main model simulation (Fig. S2). Our results show that the operating ranges of both *D* and *ω* values in our main model are constrained to satisfy the criterion of *λ* (Fig. S2E, H), indicating that, as expected, these parameters are particularly important for determining the gradient shape.

Our parameter evaluation analysis also shows that the upper and lower limits of the operating range of *K_A_* values are constrained to satisfy the [B_n_] stability criterion *g* and cortical enrichment criterion *Ratio*, respectively (Fig. S2A, C). These results suggest that, in our model, both [B_n_] stability and cortical layer enrichment are properties that are dependent on the binding/unbinding equilibrium of Bcd molecules to low-affinity DNA sites inside the nuclei. To further illustrate this point, we performed simulations using *K_A_* values that are outside the identified operating range. As shown in Fig. 5, a *K_A_* that is too large disrupts the stability of [B_n_] profiles (Fig. 5A); it also slows down Bcd diffusion to reduce the length constant *λ* (Fig. 5A). In contrast, a *K_A_* that is too small prevents Bcd molecules from being enriched in the cortical layer (Fig. 5B). At *D* and *ω* parameter values used in our main model, a systematic variation of *K_A_* identifies an operating range that satisfies all three criteria (∼3.3×10^5^ to ∼1.2×10^6^ M^−1^). These *K_A_* values correspond to a dissociation constant *K_D_* range of ∼8×10^−5^ to ∼3×10^−6^ M for non-specific DNA binding by Bcd, values that are fully consistent with the biochemical properties of homeodomains [45], [46]. We note that the estimated [DNA_sites_]_10_ value of ∼3×10^−7^ M is most likely an underestimation of the true concentration of the low-affinity sites at nuclear cycle 10 because our calculation assumes that these sites are homogeneously distributed within the entire cortical layer as opposed to the nuclear volume and, furthermore, it is likely that there are other types of low-affinity DNA sites for Bcd in addition to the 5′-TAAT-3′ sites used in our estimates. In our model, both [DNA_sites_]_10_ and *K_A_* affect the system through the equilibrium ratio of bound to free Bcd molecules within the cortical layer at nuclear cycle 10 (Eqs. 2 and 4). This ratio has an operating range between ∼0.1 and ∼0.36 that is capable of yielding Bcd gradient profiles satisfying all three of the established criteria in our main model. We note that a separate consideration of [DNA_sites_]_10_ and *K_A_* offers an opportunity to directly evaluate the biological reasonableness of the parameter values used in our simulations. It also allow us to treat embryos from different species based on their distinct physical properties to investigate interspecies scaling of the Bcd gradient profiles (unpublished).

### Evaluating the effects of mitosis on model performance

In our main model, the enrichment of Bcd molecules within the cortical layer and effective *D* are governed by the binding/unbinding chemical equilibrium of Bcd molecules to low-affinity DNA sites throughout the *Drosophila* genome. This model is consistent with previous experimental findings that the DNA-binding homeodomain itself plays a critical role in nuclear localization [47]. Our main model assumes that the disappearance of the nuclear structure at the mitotic phase does not disrupt the chemical equilibrium and, therefore, it does not consider the mitotic process separately. To specifically evaluate the effects of mitosis, we conducted simulations by incorporating a 3-minute mitotic process during which all Bcd molecules are allowed to diffuse freely (as a result of chromosome condensation that prevents Bcd from binding to non-specific DNA sites) throughout the entire embryo, thus *D_eff_*  = *D*. In these simulations, all the parameter values are identical to those used in our main model. Our results show that proper Bcd gradient profiles, as judged by the three criteria (see Fig. S3 legend for values), can be obtained when the mitotic process is specifically incorporated into our model without requiring further parameter adjustments (Fig. S3; also see Fig. S2 for a model performance comparison).

### Analyzing the effects of bcd mRNA distribution dynamics

**Figure 6:**
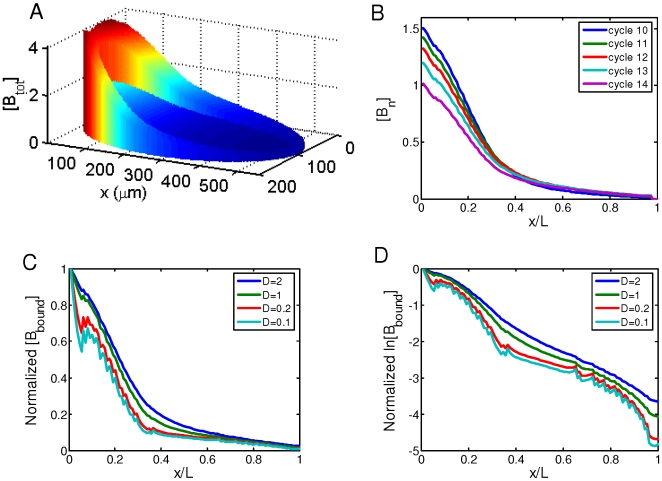
Simulating the effects of the dynamics of *bcd* mRNA distributions. A. A simulated embryo at nuclear cycle 14 showing the [B_tot_] (arbitrary units). Although the profiles of the redistributed *bcd* mRNA within the cortical layer have been reported [31], their precise functions have not been well characterized. Here we approximate the redistributed *bcd* mRNA profile during nuclear cycles 10–14 using two phases of decreases along the projected A–P axis (from the anterior, the relative *bcd* mRNA concentration within the cortical layer decreases linearly to 0.15 at *x*/*L* = 0.3 and further drops linearly to 0 at *x*/*L* = 1). Parameter values for simulations described in this figure are: *D* = 2 µm^2^s^−1^, *ω* = 0.0005 s^−1^, *K_A_* = 2×10^5^ M^−1^, and [DNA_sites_]_10_ = 3×10^−7^ M. B. Same simulation as in A, showing the stability of [B_n_] (arbitrary units) within the cortical layer during nuclear cycles 10–14. C. Same as in B, except with a progressive reduction (at selected intervals shown for clarity) of diffusion constant *D* (2, 1, 0.2, and 0.1 µm^2^s^−1^) to determine the role of Bcd protein diffusion in gradient formation. [B_bound_] within the cortical layer is normalized to 1 at *x*/*L* = 0 to facilitate the comparison between results obtained under different simulation conditions. D. Same as in C, except the normalized [B_bound_] is on ln scale. Linearity of ln[B_bound_] indicates an exponential Bcd protein gradient. See Fig. S4A for a plot of Adjusted *R* ^2^ values as a function of *D*.

A recent study challenges the diffusion model of Bcd gradient formation, raising the question of whether this process is dependent on Bcd protein diffusion [31]. We used our 2-D simulation model to specifically evaluate the effects of *bcd* mRNA dynamics on Bcd gradient formation. Our simulations are based on the available experimental data [31] and assume that *bcd* mRNA is distributed uniformly within a sphere located in the anterior during nuclear cycles 1–9 as in our main model but as a gradient-like profile within the cortical layer during nuclear cycles 10–14 (see Fig. 6 legend for further details). Since there is currently insufficient knowledge about the mechanisms leading to *bcd* mRNA redistribution, our simulations simple assume that such redistribution takes place at the onset of nuclear cycle 10 without modeling specifically this process. Fig. 6A shows the local total Bcd concentration [B_tot_] in a simulated embryo with redistributed *bcd* mRNA [31] and a diffusion constant value (*D* = 2 µm^2^s^−1^) identical to that used in Fig. 1. The simulated profiles of nuclear Bcd concentrations [B_n_] remain stable during nuclear cycles 10–14 (Fig. 6B) and exhibit an exponential function (Fig. 6D, blue line; also see below). The length constant *λ* of the nuclear Bcd profiles at nuclear cycle 14 in these simulations is 145 µm; while this value is somewhat higher than experimental measurements, those at earlier nuclear cycles are smaller and closer to experimental values (107, 124, 134 and 141 µm at nuclear cycles 10–13, respectively). These results show that, with modest parameter adjustments, our model can yield, from the redistributed *bcd* mRNA profiles, nuclear Bcd concentration profiles and dynamics properties that are broadly consistent with experimental observations.

To specifically determine whether Bcd protein diffusion might play a role in proper gradient formation, we reduced *D* progressively in our simulations. Our results show that as *D* is reduced, [B_bound_] profiles at nuclear cycle 14 become progressively less exponential (Fig. 6C,D; profiles are shown only at selected *D* values for clarity). The Adjusted *R* ^2^ values of the exponential fit (in the region of *x*/*L* = 0.2 to 0.7) decreased from 0.9854 at *D* = 2 µm^2^s^−1^, to 0.9708 and 0.8987 at *D* = 1 and 0.1 µm^2^s^−1^, respectively (see Fig. 6 legend for further details). Fig. S4A shows the Adjusted *R* ^2^ values as a function of *D* for both the main model and the model with *bcd* mRNA redistribution. While Adjusted *R* ^2^ is insensitive to *D* in our main model, this value precipitously drops as *D* decreases in the mRNA redistribution simulation. These results show that, in our model, Bcd protein diffusion is important for the formation of an exponential Bcd protein gradient. Our simulation results reveal two effects of the cortical localization of *bcd* mRNA: 1) it enhances the enrichment of Bcd molecules within the cortical layer relative to the inner part of the embryo (Fig. S4B), and 2) it enlarges the range of *D* that satisfies the stability criterion *g* (Fig. S4C). These findings provide potential biological roles of the recently reported dynamic redistribution of *bcd* mRNA.

### Evaluating nuclear size changes between different nuclear cycles

In our main model, the size of the nuclei is assumed to be a constant between different nuclear cycles, hence the relationship [B_n_]
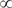
[B_bound_]/nuclear number. Furthermore, our main model assumes that the thickness of the cortical layer remains as a constant between different nuclear cycles. To determine whether changes in nuclear volume and cortical layer may affect the [B_n_] stability, we performed simulations where both the cortical layer thickness and nuclear size vary between nuclear cycles. In these simulations, [B_n_] calculations were based on estimated nuclear diameter of 10, 10.5, 9.2, 8.2 and 6.5 µm for nuclear cycles 10–14, respectively [24]. Fig. S5B and S5C show, respectively, the simulated [B_bound_] and [B_n_] profiles at different nuclear cycles (also see Fig. S5A for a 3-D presentation of [B_tot_] in the simulated embryo). These results show that our model, with proper parameter adjustments, can produce stable [B_n_] profiles when incorporating changes in both nuclear size and cortical layer thickness between different nuclear cycles (see Fig. S5 legend for further details).

### Further model evaluation and discussion

The 2-D simulation model described in this report captures key properties of the Bcd gradient system in a biologically realistic manner. This model considers changes in both nuclear number and their relative locations in early embryos. Consistent with experimental findings [24], our simulated results show that the profiles of nuclear Bcd concentrations [B_n_] are stable during nuclear cycles 10–14 (Fig. 2B). Our 2-D model also reproduces the scaling properties of the Bcd gradient in *Drosophila* embryos with a simple assumption that the amount of *bcd* mRNA (thus/or the rate of Bcd production) is proportional to the embryo volume. Our simulation results (Fig. 3) are consistent with the experimentally-observed scaling properties the Bcd gradient [7]. Finally, our model allows us to specifically evaluate other biological features of the system, including the mitotic process and *bcd* mRNA redistribution. Our results show that formation of an exponential Bcd gradient is dependent on Bcd protein diffusion in our model (Fig. 6). They also offer potential biological roles of the recently observed dynamic redistribution of *bcd* mRNA (Fig. S4).

A recent model [48] based on “nuclear trapping” of Bcd molecules has also successfully recapitulated the stability of the nuclear Bcd gradient during development. While both the nuclear trapping model and our current model support an important role of nuclei in Bcd gradient formation [24], [44], they differ from each other in the following fundamental aspects. First, the Coppey et al. model assumes that there is no Bcd-protein degradation in early embryos. Although the mechanisms controlling Bcd stability remain elusive, the experimentally observed rapid disappearance of Bcd after cellularization [9] indicates that Bcd is subject to active protein degradation in early embryos. Our model, while specifically considering Bcd degradation, is able to recapitulate the experimentally demonstrated stability of [B_n_] profiles during development (see Fig. S2G–I for the effects of *ω* on the three established criteria, with Fig. S2H showing the operating range of *ω* that satisfies the criterion of *λ*). Second, while the Coppey et al. model assumes a nuclear barrier-mediated equilibrium between Bcd molecules inside and outside the nucleus, our main model assumes a chemical equilibrium between free Bcd molecules and those that are bound to non-specific DNA sites inside the nucleus. Since nuclear localization of a protein is insufficient for proper gradient formation in embryos [44], it is evident that additional properties of Bcd, such as its DNA binding function as modeled in our study, are likely to play an important role in this process. Finally, unlike the Coppey et al. model, our model does not restrict Bcd protein diffusion in the inner part (yolk) of the embryo during nuclear cycles 10–14. It has been observed experimentally that the yolk nuclei, similar to those in the cortical layer, are also enriched with Bcd molecules [24]. We conducted simulations in which a fraction of the nuclei at the onset of nuclear cycle 10 remain in the inner part of the embryo, and we observed an enrichment of Bcd molecules in these “yolk nuclei” as seen experimentally (data not shown).

The 2-D model described in this report represents a simple and versatile tool to simulate the Bcd gradient system in a biologically realistic way. In contrast to a recent model that is also sufficient to explain [B_n_] stability [33], our simulation model takes a distinct approach and requires significantly fewer physical quantities that should be experimentally measurable and verifiable. Effectively, the biologically realistic properties of the Bcd gradient achieved in our simulations are dependent only on three relevant parameters: the diffusion constant of free Bcd molecules in the cytoplasm, the degradation rate of Bcd molecules, and the fraction of bound to free Bcd molecules at nuclear cycle 10 (see above; also see Eq. 2). While the fact that a complex biological system can be modeled with only three effective parameters illustrates the effectiveness and simplicity of our model, the choice of a limited number of parameters also poses inevitable limitations. For example, our current model does not consider the precise mechanisms of nuclear-cytoplasmic shuttling of the Bcd molecules, which is likely to be affected by the structures of nuclear pores and/or cytoplasmic islands [24], [49], [50], [51] and may be important for finer aspects of the system's behavior. We emphasize that, since our current model can recapitulate the key biological properties of the system, these three effective parameters likely represent the principal physical quantities most relevant to the system behavior.

In addition to the biological features evaluated in the current study, the versatility of our simulation model should allow us in the future to evaluate the effects of other biological features and physical or genetic perturbations on Bcd gradient formation, such as *bcd* mRNA localization [29], [52], [53], Bcd protein diffusion or degradation, or developmental clock distortions [5], [54], [55]. As an example, we have recently identified distinct features of the Bcd gradient profiles on the dorsal and ventral sides of the embryo (unpublished results). Our 2-D simulation model based on simple geometric asymmetry of the embryo can fully recapitulate our experimentally observed differences of the Bcd gradient profiles (unpublished results), differences that cannot be adequately modeled by 1-D simulations. Our simple but biologically realistic 2-D model represents a useful platform that should facilitate the formulation of experimentally testable hypotheses. It will expand the toolbox available to our studies of the fundamental mechanisms of developmental processes.

## Methods

See the Model section in Results and Discussion for details about methods.

## Supporting Information

Figure S1 Comparison between simulated data and experimental data. A. Shown are simulated [B_n_] at nuclear cycle 14 (red and green) and experimentally observed Bcd gradient profile also at early nuclear cycle 14 (blue). The experimental data shown here are from He et al. [7], which represent background-subtracted Bcd intensities, with error bars (standard deviation) shown. The two simulated [B_n_] profiles are obtained from simulations identical to the main model simulations expect the center coordinate of bcd mRNA was fine tuned to yield a [B_n_] profile that matches the experimentally observed Bcd profile (50 µm, 0; red) or the bcd mRNA is restricted to a single cube at the anterior tip of the embryo (0, 0; green). The Adjusted R2 values for our experimental data within the fitting ranges of x/L = 0.2 to 0.7 and 0 to 0.7 are 0.9961 and 0.9851, respectively. We note that both simulated Bcd profiles have lower levels in the posterior part of the embryo than the experimentally observed profile, a difference whose biological relevance will require further experimental and modeling investigations. B. Same as in A, except on ln scale. While the simulated red profile matches well with the experimental data and exhibits the experimentally observed anterior “deviation,” the simulated green profile clearly fails to exhibit this property.
(0.51 MB TIF)

Figure S2 Evaluating the effects of parameter values in different simulations. Parameter values for KA (panels A–C), D (panels D–F) and ω (panels G–I) are systematically altered to evaluate model performance. Three criteria are used in these evaluations: [B_n_] stability as measured by g (panels A, D and G), gradient shape as measured by length constant λ at nuclear cycle 14 (panels B, E, H), and cortical enrichment as measured by the ratio of total Bcd molecules in the cortical layer to those in the inner part of the embryo at nuclear cycle 14 (panels C, F and I). The regions where the Bcd gradient profiles satisfy the established criteria, i.e., |g|<0.1, Ratio>1.5, and 1.5<λ/L<2.0, are shaded. In these analyses, individual parameters are systematically changed in simulations when the other two parameters are at set values at their respective model simulations. Different colors represent different simulation procedures as indicated in the figure.
(0.76 MB TIF)

Figure S3 2-D simulation with mitosis. A. A simulated embryo at nuclear cycle 14 showing [B_tot_] (arbitrary units). In this simulation, the mitotic process is specifically considered at nuclear cycles 10–13, during which all Bcd molecules are allowed to diffuse freely. Other parameter values are identical to those used in the main model. The A–P position is shown as absolute distance x (in µm) from the anterior. At nuclear cycle 14, the ratio of total Bcd molecules in the cortical layer to those in the inner part of the embryo is 1.7975. B. A plot of [B_bound_] (arbitrary units) within the cortical layer as a function of x/L, at nuclear cycles 10–14. C. Same as in B, except now showing [B_n_] within the cortical layer at nuclear cycles 10–14. Similar to the main model (Fig. 2B), the mitotic process does not affect [B_n_] stability (g = 0.0143). The simulated [B_n_] profile at nuclear cycle 14 has a length constant λ = 93.3 µm. See Fig. S2 for a model performance comparison.
(0.73 MB TIF)

Figure S4 Investigating the effects of bcd mRNA redistribution. A. Adjusted R2 value of the exponential fitting (within the range of x/L = 0.2 to 0.7) of simulated [B_bound_] is plotted as a function of D. For comparative purposes, results obtained from both the main model simulation (red) and the simulation with bcd mRNA redistribution (blue) are shown. Note the difference in Adjusted R2 sensitivity to D. B. The ratio of total Bcd molecules in the cortical layer to those in the inner part of the embryo plotted as a function of D. Color codes are the same as in A. Regions where Ratio >1.5 are shaded. Note the higher Ratio value obtained in the mRNA redistribution simulation (blue). C. A plot of g as a function of D, with color codes being the same as in A. Regions where |g|<0.1 are shaded. Note the blue curve is within the shaded area under all D values tested, suggesting another potential role of the observed bcd mRNA redistribution.
(0.54 MB TIF)

Figure S5 Evaluating the effects of nuclear size and cortical layer at different nuclear cycles. A. A simulated embryo at 5 min into nuclear cycle 14 showing [B_tot_] (arbitrary units). In this simulation, non-specific DNA binding site concentrations were calculated based on the following estimates: the thickness of the cortical layer is 15, 20, 20 23, and 25 µm for nuclear cycles 10–14, respectively, corresponding to a cortical layer volume of ∼3.3, 4.2, 4.2 4.7, and 5.0 nl. The concentrations of the non-specific DNA binding sites within the cortical layer are: 5×10^−7^, 4×10^−7^, 4×10^−7^, 3.6×10^−7^, and 3.4×10^−7^ M, for nuclear cycles 10–14, respectively. The relative volumes of a single nucleus, which were calculated based on the experimental estimates [24], are 3.64, 4.21, 2.83, 2.0, and 1.0 for nuclear cycles 10–14, respectively. Parameter values used in this simulation are: D = 6 µm^2^s^−1^, ω = 0.0004 s^−1^, KA = 2.4×10^6^ M^−1^. At nuclear cycle 14, the ratio of total Bcd molecules in the cortical layer to those in the inner part of the embryo is 5.0044. B. A plot of [B_bound_] (arbitrary units) within the cortical layer as a function of x/L, at nuclear cycles 10–14. C. Same as in B, except now showing nuclear Bcd concentrations [B_n_] at nuclear cycles 10–14. [B_n_] is calculated from [B_bound_] at each nuclear cycle based on the nuclear number and the volumes of each nucleus and the cortical layer. As seen in the main model (Fig. 2B), [B_n_] profiles exhibit stability between different nuclear cycles (g = −0.050) when changes in nuclear volume and cortical layer between different nuclear cycles are incorporated in our simulation. The [B_n_] profile at nuclear cycle 14 has a length constant λ = 105 µm. We note that the results shown in Fig. S5 do not represent an improvement over those obtained in the main model simulations. It is possible that the use of additional parameters may improve the simulation outcome when more biological features are included in our model.
(0.75 MB TIF)
