# Supplementary figures and images for: A Two-Dimensional Simulation Model of the Bicoid Gradient in Drosophila
Source: PLoS One. 2010 Apr 21;5(4):e10275. doi: 10.1371/journal.pone.0010275 (PMC2858077; doi:10.1371/journal.pone.0010275)

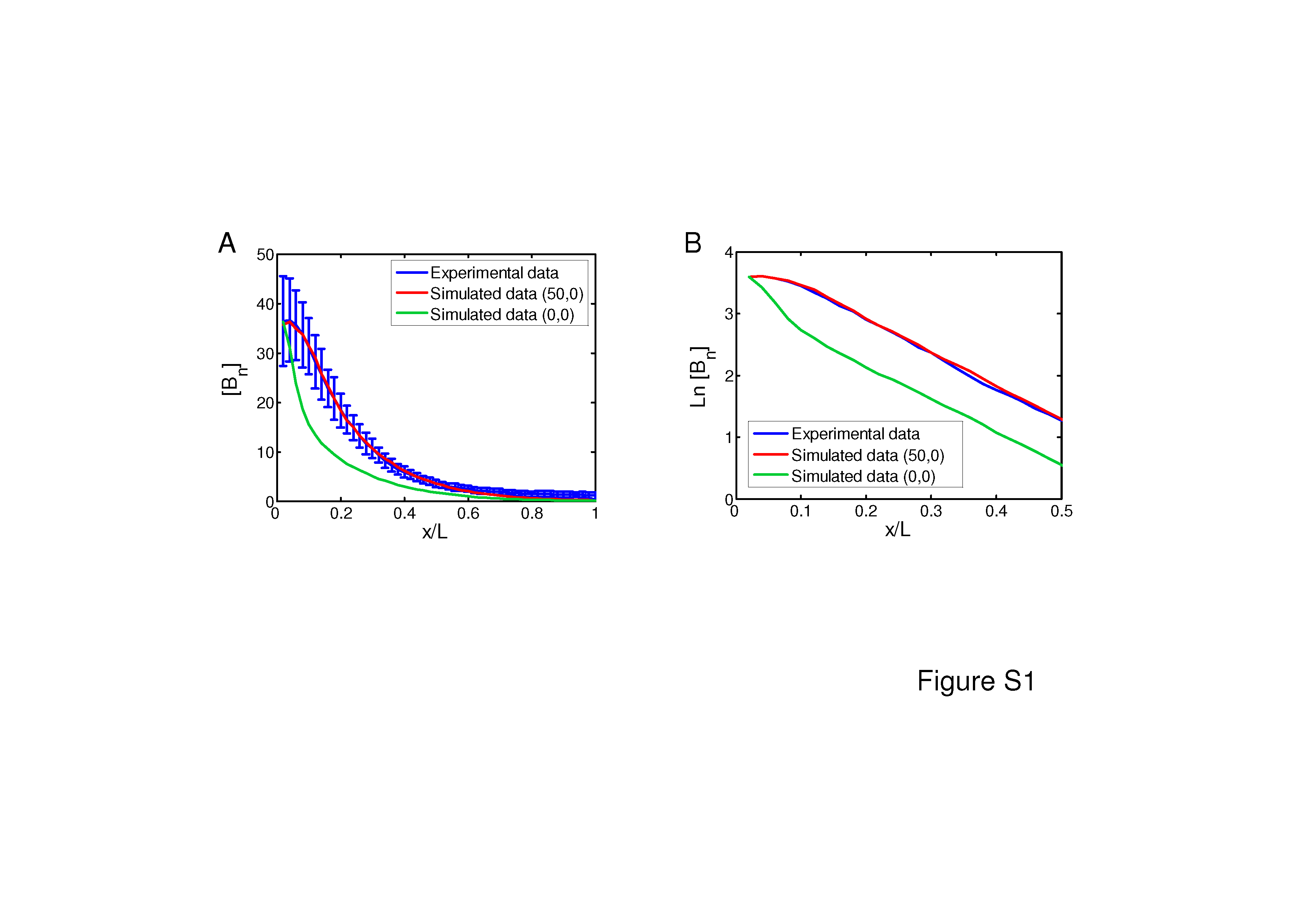

Supplement: Figure S1 — Comparison between simulated data and experimental data. A. Shown are simulated [Bn] at nuclear cycle 14 (red and green) and experimentally observed Bcd gradient profile also at early nuclear cycle 14 (blue). The experimental data shown here are from He et al. [7], which represent background-subtracted Bcd intensities, with error bars (standard deviation) shown. The two simulated [Bn] profiles are obtained from simulations identical to the main model simulations expect the center coordinate of bcd mRNA was fine tuned to yield a [Bn] profile that matches the experimentally observed Bcd profile (50 µm, 0; red) or the bcd mRNA is restricted to a single cube at the anterior tip of the embryo (0, 0; green). The Adjusted R2 values for our experimental data within the fitting ranges of x/L = 0.2 to 0.7 and 0 to 0.7 are 0.9961 and 0.9851, respectively. We note that both simulated Bcd profiles have lower levels in the posterior part of the embryo than the experimentally observed profile, a difference whose biological relevance will require further experimental and modeling investigations. B. Same as in A, except on ln scale. While the simulated red profile matches well with the experimental data and exhibits the experimentally observed anterior “deviation,” the simulated green profile clearly fails to exhibit this property. (0.51 MB TIF) [file pone.0010275.s001.tif]

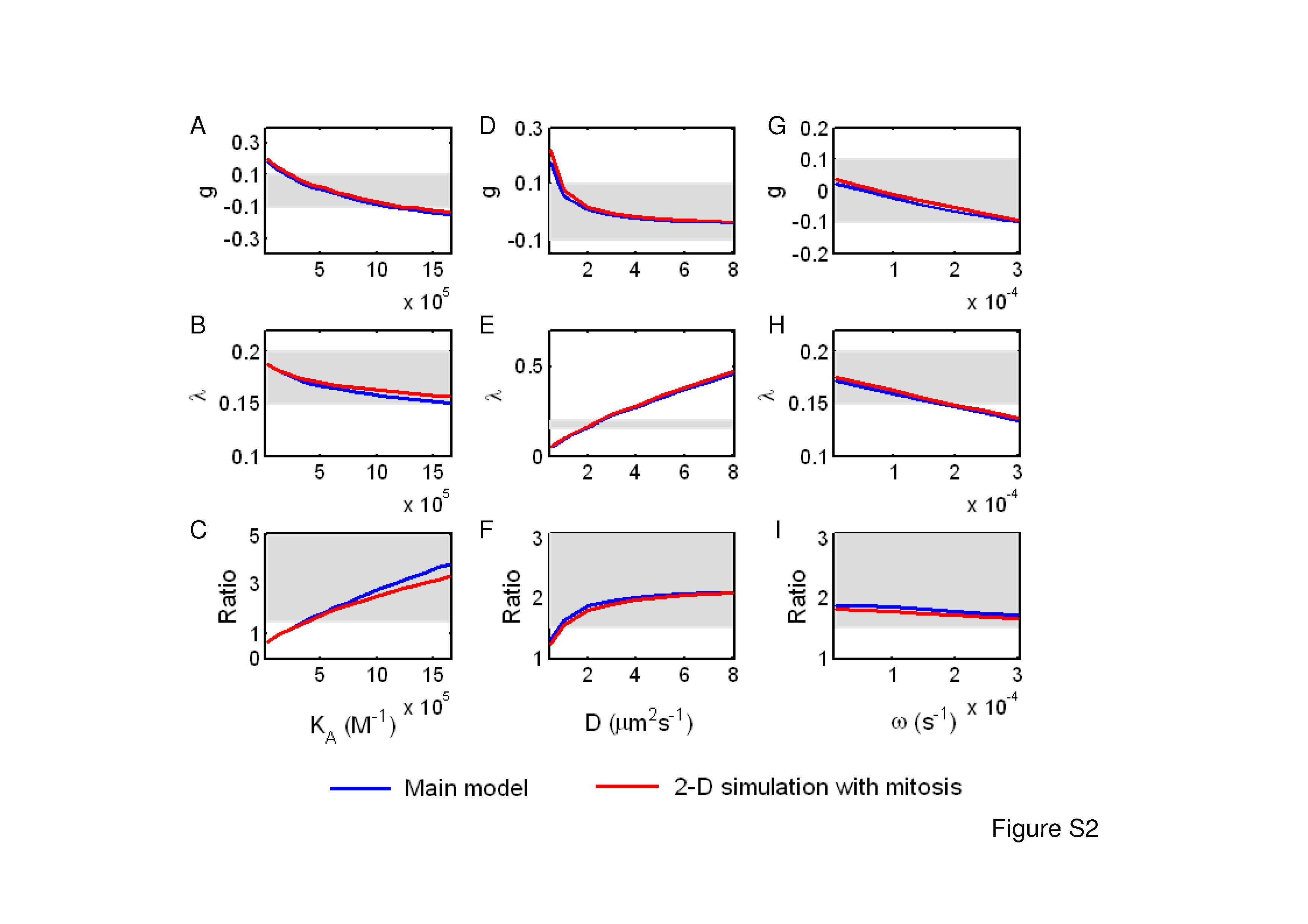

Supplement: Figure S2 — Evaluating the effects of parameter values in different simulations. Parameter values for KA (panels A–C), D (panels D–F) and ω (panels G–I) are systematically altered to evaluate model performance. Three criteria are used in these evaluations: [Bn] stability as measured by g (panels A, D and G), gradient shape as measured by length constant λ at nuclear cycle 14 (panels B, E, H), and cortical enrichment as measured by the ratio of total Bcd molecules in the cortical layer to those in the inner part of the embryo at nuclear cycle 14 (panels C, F and I). The regions where the Bcd gradient profiles satisfy the established criteria, i.e., |g|<0.1, Ratio>1.5, and 1.5<λ/L<2.0, are shaded. In these analyses, individual parameters are systematically changed in simulations when the other two parameters are at set values at their respective model simulations. Different colors represent different simulation procedures as indicated in the figure. (0.76 MB TIF) [file pone.0010275.s002.tif]

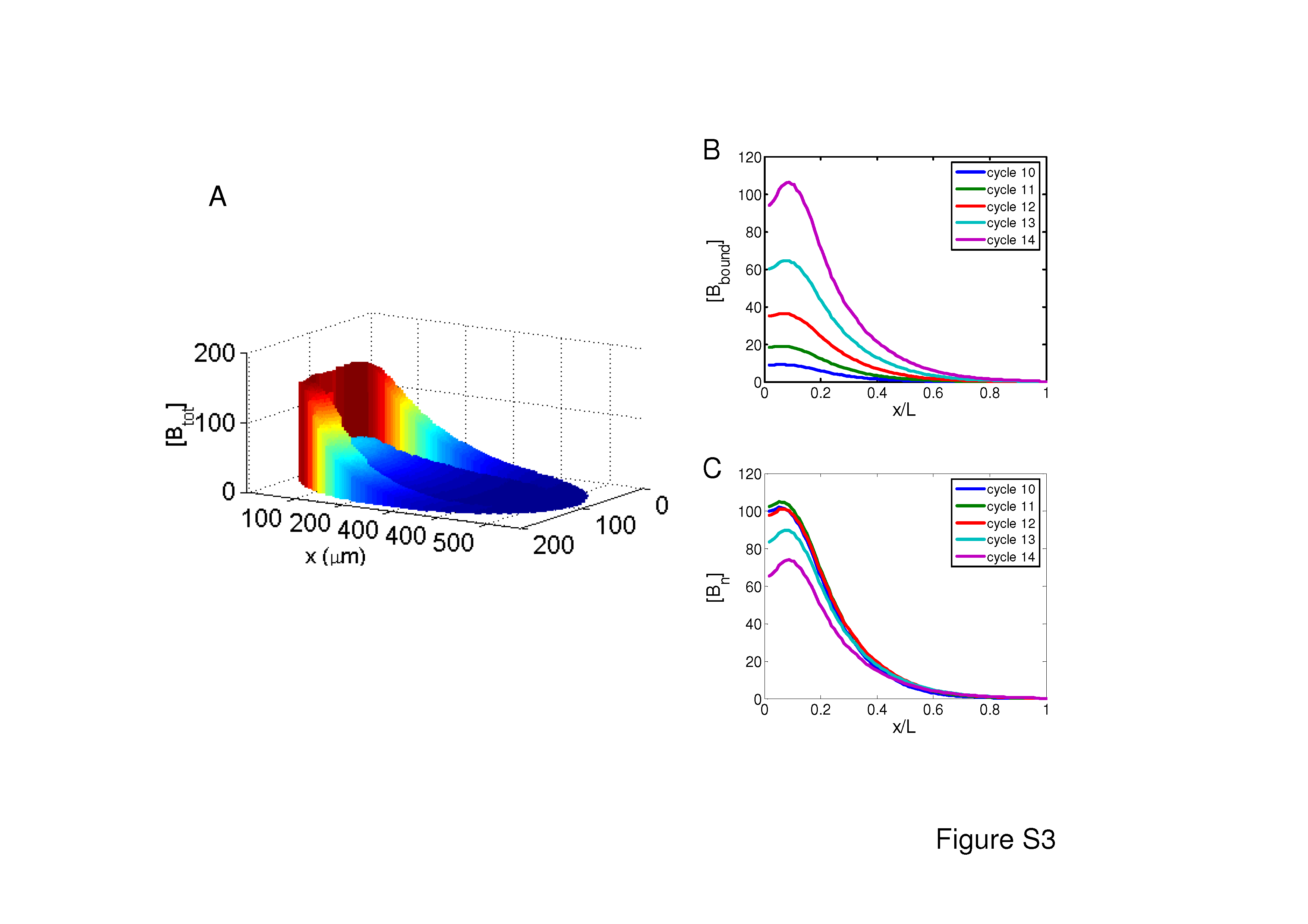

Supplement: Figure S3 — 2-D simulation with mitosis. A. A simulated embryo at nuclear cycle 14 showing [Btot] (arbitrary units). In this simulation, the mitotic process is specifically considered at nuclear cycles 10–13, during which all Bcd molecules are allowed to diffuse freely. Other parameter values are identical to those used in the main model. The A–P position is shown as absolute distance x (in µm) from the anterior. At nuclear cycle 14, the ratio of total Bcd molecules in the cortical layer to those in the inner part of the embryo is 1.7975. B. A plot of [Bbound] (arbitrary units) within the cortical layer as a function of x/L, at nuclear cycles 10–14. C. Same as in B, except now showing [Bn] within the cortical layer at nuclear cycles 10–14. Similar to the main model (Fig. 2B), the mitotic process does not affect [Bn] stability (g = 0.0143). The simulated [Bn] profile at nuclear cycle 14 has a length constant λ = 93.3 µm. See Fig. S2 for a model performance comparison. (0.73 MB TIF) [file pone.0010275.s003.tif]

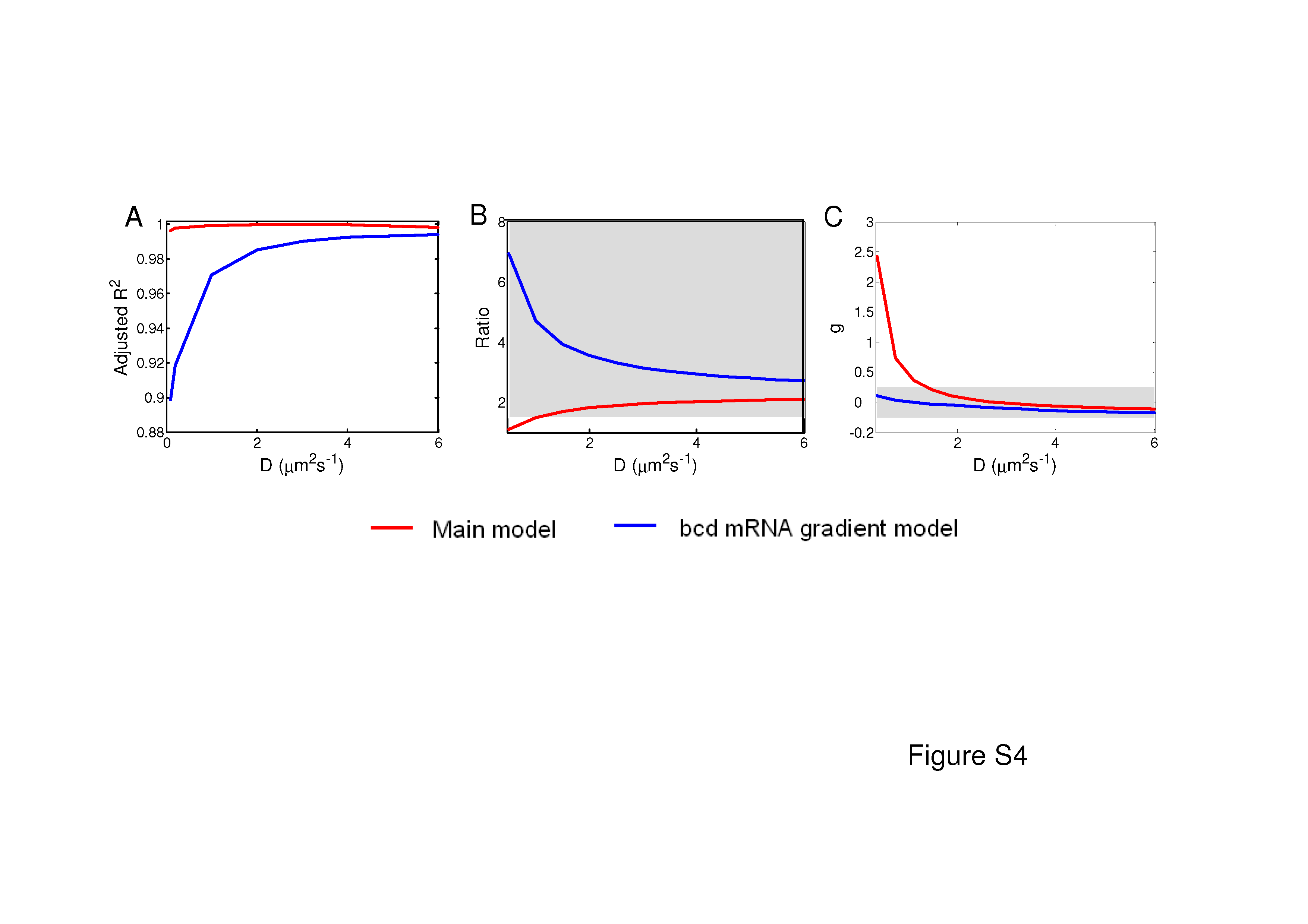

Supplement: Figure S4 — Investigating the effects of bcd mRNA redistribution. A. Adjusted R2 value of the exponential fitting (within the range of x/L = 0.2 to 0.7) of simulated [Bbound] is plotted as a function of D. For comparative purposes, results obtained from both the main model simulation (red) and the simulation with bcd mRNA redistribution (blue) are shown. Note the difference in Adjusted R2 sensitivity to D. B. The ratio of total Bcd molecules in the cortical layer to those in the inner part of the embryo plotted as a function of D. Color codes are the same as in A. Regions where Ratio >1.5 are shaded. Note the higher Ratio value obtained in the mRNA redistribution simulation (blue). C. A plot of g as a function of D, with color codes being the same as in A. Regions where |g|<0.1 are shaded. Note the blue curve is within the shaded area under all D values tested, suggesting another potential role of the observed bcd mRNA redistribution. (0.54 MB TIF) [file pone.0010275.s004.tif]

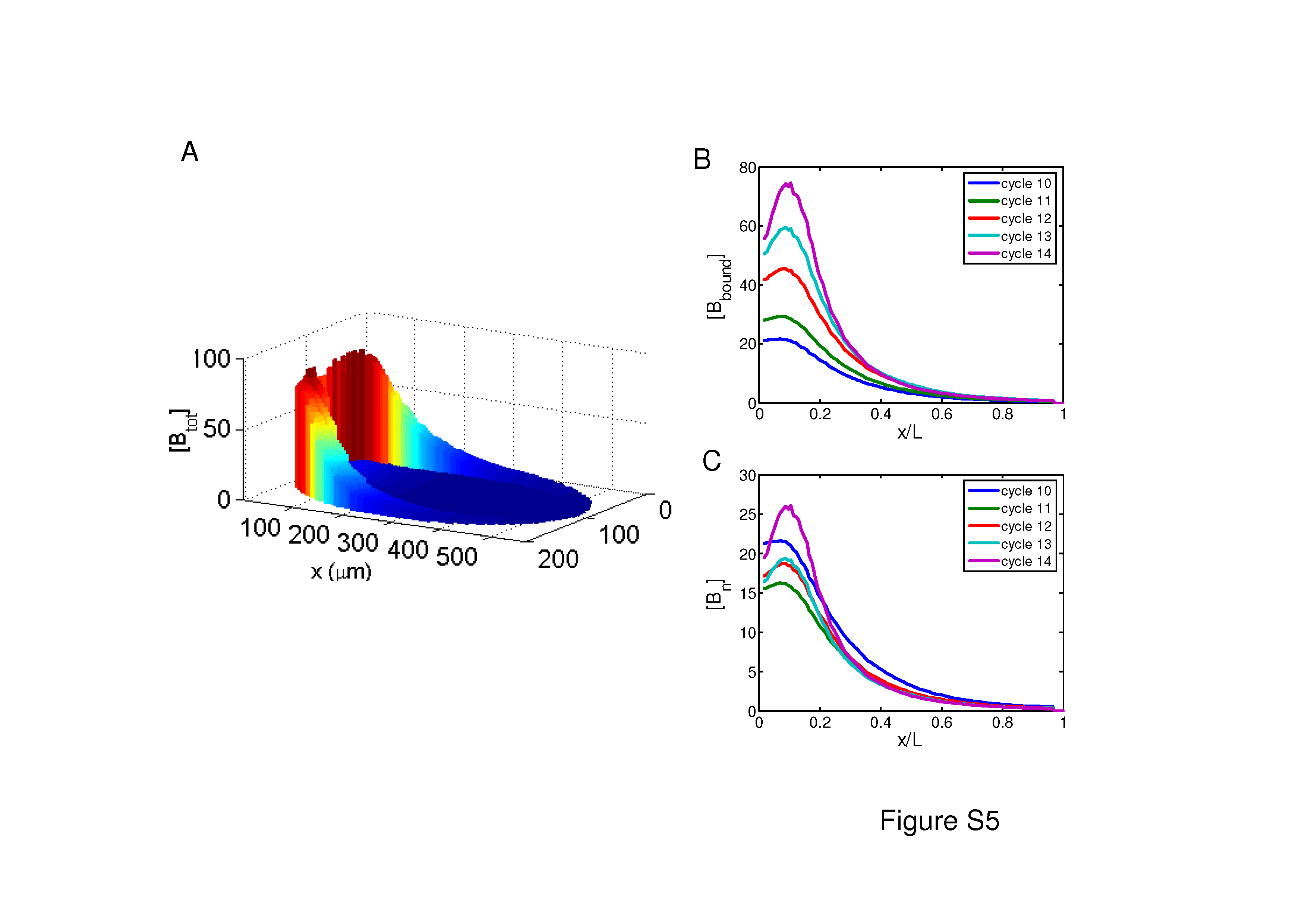

Supplement: Figure S5 — Evaluating the effects of nuclear size and cortical layer at different nuclear cycles. A. A simulated embryo at 5 min into nuclear cycle 14 showing [Btot] (arbitrary units). In this simulation, non-specific DNA binding site concentrations were calculated based on the following estimates: the thickness of the cortical layer is 15, 20, 20 23, and 25 µm for nuclear cycles 10–14, respectively, corresponding to a cortical layer volume of ∼3.3, 4.2, 4.2 4.7, and 5.0 nl. The concentrations of the non-specific DNA binding sites within the cortical layer are: 5×10^−7^, 4×10^−7^, 4×10^−7^, 3.6×10^−7^, and 3.4×10^−7^ M, for nuclear cycles 10–14, respectively. The relative volumes of a single nucleus, which were calculated based on the experimental estimates [24], are 3.64, 4.21, 2.83, 2.0, and 1.0 for nuclear cycles 10–14, respectively. Parameter values used in this simulation are: D = 6 µm2s−1, ω = 0.0004 s−1, KA = 2.4×10^6^ M−1. At nuclear cycle 14, the ratio of total Bcd molecules in the cortical layer to those in the inner part of the embryo is 5.0044. B. A plot of [Bbound] (arbitrary units) within the cortical layer as a function of x/L, at nuclear cycles 10–14. C. Same as in B, except now showing nuclear Bcd concentrations [Bn] at nuclear cycles 10–14. [Bn] is calculated from [Bbound] at each nuclear cycle based on the nuclear number and the volumes of each nucleus and the cortical layer. As seen in the main model (Fig. 2B), [Bn] profiles exhibit stability between different nuclear cycles (g = −0.050) when changes in nuclear volume and cortical layer between different nuclear cycles are incorporated in our simulation. The [Bn] profile at nuclear cycle 14 has a length constant λ = 105 µm. We note that the results shown in Fig. S5 do not represent an improvement over those obtained in the main model simulations. It is possible that the use of additional parameters may improve the simulation outcome when more biological features are included in our model. (0.75 MB TIF) [file pone.0010275.s005.tif]
